# The associations of diet quality and cardiometabolic indicators in children and the mediation role of cardiorespiratory fitness

**DOI:** 10.3389/fnut.2025.1632493

**Published:** 2025-10-23

**Authors:** Ping-Ping Zhang, Gao-Feng Lin, Jia-Ying Gu, Bi-Lian Wang, Jie Zhang, Ye Zhou, Miao Xu, Hui Wang, Li Li

**Affiliations:** ^1^Department of Endocrinology and Metabolism, The First Affiliated Hospital of Ningbo University, Ningbo, Zhejiang, China; ^2^Ningbo Center for Healthy Lifestyle Research, Chronic Disease Management Office, The First Affiliated Hospital of Ningbo University, Ningbo, Zhejiang, China; ^3^Fuming Street Community Health Service Center of Yinzhou District, Ningbo, Zhejiang, China; ^4^School Medical Office, Ningbo Art Experimental School, Ningbo, Zhejiang, China; ^5^Health Science Center, Ningbo University, Ningbo, Zhejiang, China; ^6^Department of Maternal and Child Health, School of Public Health, Peking University, Beijing, China

**Keywords:** children, diet quality, cardiometabolic risk, cardiorespiratory fitness, mediation effect

## Abstract

**Background:**

Cardiometabolic health in children has become a growing global concern due to its long-term association with chronic diseases such as cardiovascular disease and type 2 diabetes. Diet quality plays a critical role in determining cardiometabolic health. This study explored the association between diet quality and cardiometabolic health indicators in Chinese children aged 8–10 years and assessed the mediating role of cardiorespiratory fitness (CRF).

**Methods:**

A total of 1,389 third-grade students from Ningbo, China were included. Diet quality was assessed using a validated questionnaire to calculate a Global Dietary Recommendations (GDR) score, with higher scores indicating healthier dietary patterns. Anthropometric data and fasting blood samples were collected to evaluate metabolic markers. A cardiometabolic risk (CMR) score was calculated based on age- and sex-adjusted Z-scores for waist circumference, systolic blood pressure, triglycerides, total cholesterol to high-density lipoprotein cholesterol ratio, and homeostasis model assessment for insulin resistance (HOMA-IR). CRF was measured using the 20-meter shuttle run test. Generalized linear mixed models were used to examine associations, and mediation analysis was conducted to assess the mediating effect of CRF.

**Results:**

After adjusting for age, sex, and physical activity, higher GDR scores were significantly associated with lower fasting insulin (*β* = −0.013; *p* = 0.023), lower HOMA-IR (*β* = −0.014; *p* = 0.019), and reduced CMR score (*β* = −0.074; *p* = 0.030). Stratified and interaction analyses revealed stronger associations in girls than in boys. Mediation analysis showed that CRF accounted for 26.1% of the association between GDR score and fasting insulin, 25.3% for HOMA-IR, and 32.9% for CMR score (all *p* < 0.05).

**Conclusion:**

In Chinese children, better diet quality is associated with more favorable cardiometabolic profiles. CRF showed a statistically significant mediating role in the cross-sectional association, highlighting the potential importance of both healthy eating and physical fitness in promoting cardiometabolic health in youth.

## 1 Introduction

Childhood obesity has become a global epidemic (1), one of the key consequences is the rise in cardiometabolic risk (CMR) (2, 3), CMR is commonly defined by a combination of factors including central obesity, dyslipidemia, elevated blood pressure, and impaired glucose metabolism (4). Lifestyle-related determinants such as diet and physical activity habits may contribute to establishing CMR (5, 6). Among these, dietary quality has emerged as a major determinant, poor diet have been demonstrated to play a significant role in the development of chronic diseases including diabetes, cardiovascular disease, stroke, certain cancers, and obesity, leading to a large proportion of morbidity and early deaths (7). Globally, poor diet is the second leading risk factor for non-communicable diseases (8).

Diet quality refers to the extent to which dietary patterns or food intake align with dietary guidelines, and is commonly assessed based on the quality and diversity of food consumed (9). Existing evidence suggests that higher dietary quality is associated with improved cardiometabolic outcomes (10), for instance, healthy diets, characterized by rich in fruits, vegetables, whole grains, and unsaturated fats, and low in processed foods and sugar sweetened beverage, have been linked to lower CMR in adults (11). In children, studies have demonstrated similar trends (12), though the data are often limited to specific regions or dietary patterns (13–16). Diet quality indicators serve as instruments designed to evaluate an individual’s overall diet quality, while the measurement of diet quality in pediatrics has not well established and validated (17).

The Diet Quality Questionnaire (DQQ) is a low-burden tool and was constructed using 29 food groups to capture population-level dietary patterns through simple yes/no questions about sentinel foods (i.e., items representing >95% consumption within each group) (18). It has been validated in Chinese children aged 7–18 years, and Global Dietary Recommendations (GDR) scores were calculated including: (1) *GDR-Healthy* (health-promoting foods), (2) *GDR-Limit* (components to restrict), and (3) *overall GDR* (composite score) (19). Notably, the DQQ’s brevity (5-min administration) and alignment with WHO guidelines make it particularly suitable for pediatric studies, evidence in Chinese youth showed that higher GDR-Limit scores correlated with increased obesity odds (OR = 1.43), while overall GDR scores showed protective effects (19). Compared to traditional methods like 24-h recalls, the DQQ balances practicality with scientific rigor (20).

Cardiorespiratory fitness (CRF), defined as the ability of the circulatory and respiratory systems to supply oxygen to skeletal muscles during sustained physical activity, is another key determinant of cardiometabolic health (21–23). CRF is typically measured by maximal oxygen uptake (VO₂max) or performance in endurance tests like the 20-meter shuttle run test (20mSRT). Higher CRF levels have been associated with enhanced insulin sensitivity, reduced systemic inflammation, improved lipid metabolism, and better cardiovascular outcomes in children and adolescents with obesity (24). Importantly, CRF is a modifiable factor that can be influenced by physical activity like high-intensity interval training (25). In children, CRF not only predicts current health status but also serves as a significant marker of future health risks (26). Emerging evidence suggests that CRF may act as a mediator between lifestyle factors (eg. sugar-sweetened beverage consumption) and adiposity (27). While the importance of CRF in this context has been recognized, the specific mechanisms through which it moderates the impact of diet quality on cardiometabolic health remain underexplored, particularly in pediatric populations.

This study had two primary objectives: first, to examine the associations between the GDR score and cardiometabolic health indicators in Chinese children and assess potential sex differences; and second, to evaluate whether CRF mediates the relationship between diet quality and cardiometabolic outcomes in this population.

## 2 Methods

### 2.1 Study participants

**Figure 1:**
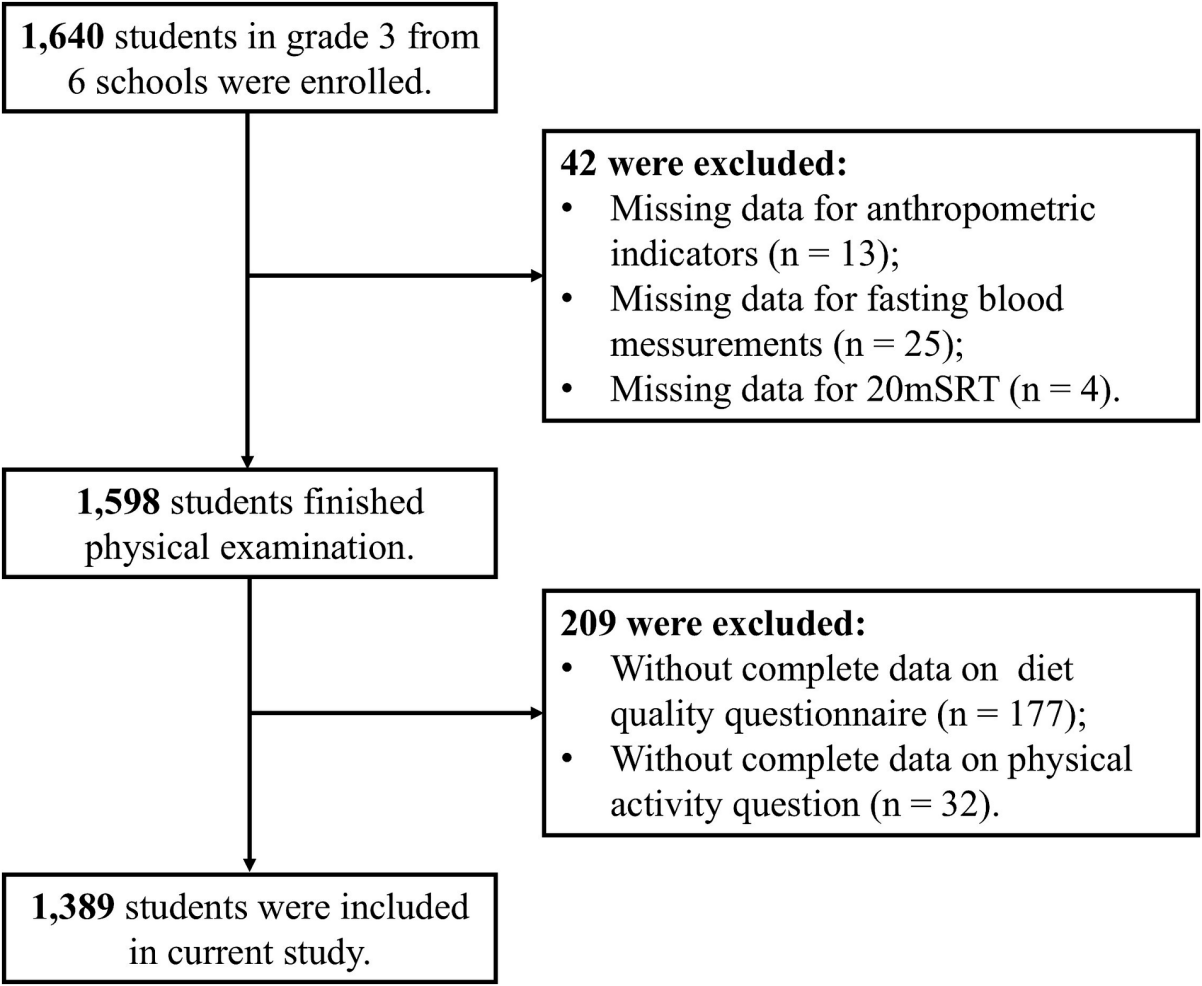
Flow chart for included participants.

This is a cross-sectional study used the baseline data of “Optimizing Intervention Effects in Children and Adolescents in Ningbo (OptiChild study)” program (28), which is a clustered randomized controlled trial (Registration No. at clinicaltrials.gov: NCT05482165). This program recruited 1,640 third-grade students between 8 to 10 years old from six primary schools in three districts of Ningbo city in September 2022. The current study used the baseline data and included 1,389 students with the flowchart of the selection of the study population showed in Figure 1.

The program received approval from the Ethics Committee of the First Affiliated Hospital of Ningbo University (Approval No. 2021-R168), and obtained written informed consent from all participating students and their primary guardians.

### 2.2 Anthropometry and biochemical measurements

All the anthropometry measurements were measured by trained staff from local community healthcare centers, adhering strictly to standardized procedures at the participants’ schools. Participant height was measured using a mechanical stadiometer, with individuals barefoot to ensure precision. Body weight and fat mass were assessed via a bioimpedance analysis system (Inbody770, California, USA), with participants dressed in lightweight attire and no footwear. Waist circumference (WC) was gauged using a non-elastic tape, placed at the midpoint between the lower rib and iliac crest, taken at the conclusion of a normal exhalation. Hip circumference was s measured at the maximal protrusion of the gluteal muscles using a non-elastic tape, with participants standing upright in lightweight clothing and feet together. All recorded values for height, weight, WC, and hip circumference were precise to within 0.1 units. Diastolic blood pressure (DBP) and systolic blood pressure (SBP) were measured using an Omron digital sphygmomanometer (Omron HEM-7121, Kyoto, Japan) after the participants had rested for at least 5 min.

Blood samples were collected from the elbow vein by certified nurse using a standardized protocol during morning health checkups at school. All samples were obtained following an overnight fast, stored at 4 °C, and analyzed on the same day. Fasting insulin (FINS) were assessed using a chemiluminescent technique on an automated analyzer (Roche Cobas E602 Immunology Analyzer, Basel, Switzerland). Levels of fasting plasma glucose (FPG), triglycerides (TG), low-density lipoprotein cholesterol (LDL-C), high-density lipoprotein cholesterol (HDL-C), and total cholesterol (TC) were determined using enzymatic assays with a separate automated system (Beckman AU5800, California, USA).

Body mass index (BMI) was determined by dividing weight (in kilograms) by the square of height (in meters). BMI Z scores were calculated to account for age and sex variations in pediatric growth patterns (29), allowing for more appropriate interpretation of BMI in children. Body fat percentage (BFP) was calculated by dividing fat mass by total body weight and multiplying by 100. The waist-to-hip ratio (WHR) was computed as waist circumference divided by hip circumference, while the waist-to-height ratio (WHtR) was obtained by dividing waist circumference by height. The homeostasis model assessment for insulin resistance (HOMA-IR) was calculated as [FINS (μU/L) * FPG (mg/dL)]/405 (30). Various approaches have been utilized to compute continuous CMR score, most of which include metrics related to adiposity, lipid profiles, metabolic markers, and blood pressure (31). In this study, CMR score were derived by adding the age- and sex-specific Z scores for WC, SBP, TG, the TC/HDL-C ratio, and HOMA-IR, which aligns with established pediatric CMR scoring methods (31, 32). The Z scores for each parameter were calculated using the formula (value − mean)/SD, separately for boys and girls within each one-year age group. Higher CMR score corresponded to an elevated cardiometabolic risks.

### 2.3 Questionnaires

All children were asked to recall their food group consumption over the previous day and night, and completed the DQQ with the assistance of a trained project investigator, who used simplified, child-friendly language to ensure understanding. Food intake was coded into 29 food groups following the DQQ tool fitted in Chinese population, which employs binary (yes/no) questions about specific food groups consumed in the past 24 h (33), and the Chinese version DQQ tool been validated for Chinese children aged 7–18 year (19). The GDR-Healthy score and GDR-Limit score have a range from 0 to 9, reflects adherence to global dietary recommendations on healthy components of the diet and components of the diet to limit or avoid, respectively. A higher GDR-Healthy score indicates inclusion of more health-promoting foods in the diet, and the GDR-Limit score is opposite. The overall GDR score was calculated as: GDR scores = GDR-Healthy - GDR-Limit + 9, ensuring a positive scale where higher values indicate better diet quality, it ranged from 0 to 18. The higher the GDR score, the more recommendations are likely to be met. All participants were divided into four quantiles based on their GDR score, the distribution across the quantiles was as follows: 219 in Q1, 397 in Q2, 425 in Q3, and 348 in Q4.

Assessment of physical activity of moderate- or vigorous-intensity physical activity (MVPA) time in school days and weekends was asked by a self-reported Physical Activity Questionnaire, which has been validated in Chinese children (34), with the details of the questionnaire described before elsewhere (35). The daily MVPA time were categorized in to ≥ 60 or < 60 min/day last week according to the WHO recommendations (36).

### 2.4 Cardiorespiratory fitness test

A qualified physical education instructor administered the CRF evaluation using the 20mSRT, a validated and reliable method for assessing CRF in children and youth (37). During the 20mSRT, participants ran back and forth between two lines set 20 meters apart, synchronizing their pace with audio cues. The test is structured into multiple stages (or levels), each lasting about 1 min and consisting of several 20-meter laps (referred to as shuttles). The initial running speed was 8.5 km/h, increasing by 0.5 km/h each minute (with 1 min corresponding to one stage) (38). The test concluded when a participant could not reach the line in time with the audio signals for two consecutive attempts or when they halted due to exhaustion. The total number of completed laps was then used as the primary indicator to estimate each child’s CRF level.

### 2.5 Statistical analysis

The demographic and physical characteristics of the participants were summarized using descriptive statistics. Normality of continuous variables was assessed using *Shapiro–Wilk* tests and visual inspection of *Q-Q* plots. Group differences across GDR score quantiles were examined using one-way analysis of variance (*ANOVA*) for normally distributed variables (reported as mean ± SD) or the *Kruskal-Wallis* test for skewed variables (reported as median [IQR]). Categorical variables were expressed as counts (n) with percentages (%), and their distribution across GDR score quantile groups was assessed using the *chi-square* test. For continuous variables with significant overall differences across GDR score quantiles, pairwise comparisons were performed using *Tukey’s post-hoc* test for normally distributed variables and *Dunn’s* test with Bonferroni correction for non-normally distributed variables. For categorical variables with significant overall differences, pairwise comparisons were performed using Bonferroni-corrected *chi-square* tests.

The relationships between GDR score and CMR indicators and the interaction terms (GDR score ×sex) were explored by a generalized linear mixed-effects model (GLMM), and the FINS, HOMA-IR and TG levels were Ln-transformed due to skewed distributions. This model incorporated school-level random intercepts to account for the clustering of participants within schools, as data collection was conducted at the school level. Two models were constructed: Model 1 was unadjusted, while Model 2 included additional adjustments for age, sex, and MVPA based on Model 1. Stratified analyses were conducted to evaluate the association of GDR score and CMR indicators among children with different gender.

To assess the mediating role of CRF in the association between the GDR score and CMR indicators, the *mediation* package in R was utilized. Bootstrapping with 5,000 resamples was performed to estimate the indirect effects. Statistical significance was determined using a two-tailed test with a *p*-value threshold of <0.05. All statistical analyses were conducted using R version 4.3.0 (R Core Team).

## 3 Results

### 3.1 Clinical, demographic and dietary characteristics stratified by GDR score quartile

A total of 1,389 participants were included in the analysis, we compared the characteristics of the children included and 251 children excluded (Supplementary Table S1), no differences were detected between the students included and excluded. The histogram of the GDR score showed a normal distribution, with values ranging from 3 to 18 (Supplementary Figure S1).

The overall sample consisted of 52.7% boys and 47.3% girls, with a mean age of 8.48 ± 0.29 years. The clinical and demographic characteristics stratified by GDR score quartiles are presented in Table 1. Significant differences were observed in sex distribution (*p <* 0.001), WHR (*p =* 0.029), FPG (*p =* 0.022), and CRF (laps, *p =* 0.020). Participants in higher GDR quantiles (Q4) exhibited greater proportions of girls, higher CRF, and lower FPG. For example, FPG levels were significantly higher in Q1 compared with Q4, whereas Q2 and Q3 were intermediate and did not differ significantly from either group. Time spent in MVPA also differed significantly across quantiles (*p <* 0.001), with Q4 showing the highest proportion of participants engaging in >1 h/day of MVPA (60.6%).

**Table 1:** Basic characteristics by quantiles of GDR score.

Variables	Q1	Q2	Q3	Q4	Overall	*p*
*n*	219	397	425	348	1,389	
Sex, *n* (%)						**<0.001**
Boys	134 (61.2)^a^	245 (61.7)^a^	209 (49.2)^b^	144 (41.4)^b^	732 (52.7)	
Girls	85 (38.8)^a^	152 (38.3)^a^	216 (50.8)^b^	204 (58.6)^b^	657 (47.3)	
Age, years	8.47 (0.29)	8.47 (0.30)	8.48 (0.29)	8.50 (0.29)	8.48 (0.29)	0.400
Anthropometric variables						
Height, cm	132.6 (5.9)	132.9 (5.6)	132.3 (5.8)	132.4 (5.5)	132.4 (5.7)	0.900
Weight, kg	29.7 (6.3)	29.4 (6.2)	29.5 (6.5)	28.8 (5.7)	29.3 (6.2)	0.338
Waist circumference, cm	57.9 (7.2)	57.7 (7.0)	57.8 (7.1)	56.9 (6.6)	57.6 (7.0)	0.273
Hip circumference, cm	70.4 (6.6)	70.5 (6.5)	70.7 (6.9)	70.2 (6.2)	70.5 (6.6)	0.195
WHR	0.82 (0.05)^a^	0.82 (0.05)^ab^	0.82 (0.05)^ab^	0.81 (0.05)^b^	0.82 (0.05)	**0.029**
WHtR	0.44 (0.04)	0.44 (0.05)	0.44 (0.05)	0.43 (0.05)	0.43 (0.05)	0.131
BFP, %	20.0 (7.5)	20.0 (8.1)	20.4 (8.1)	19.6 (7.8)	20.0 (7.9)	0.533
BMI *Z* score	0.28 (1.26)	0.20 (1.27)	0.24 (1.36)	0.07 (1.21)	0.19 (1.28)	0.195
BMI, kg/m^2^	16.8 (2.6)	16.6 (2.6)	16.7 (2.8)	16.4 (2.5)	16.6 (2.6)	0.191
Blood pressure						
SBP, mmHg	101.7 (10.6)	101.3 (10.4)	101.7 (10.6)	101.6 (10.0)	101.6 (10.4)	0.927
DBP, mmHg	63.8 (7.9)	63.0 (7.3)	63.4 (6.9)	63.2 (8.3)	63.3 (7.6)	0.561
Biochemical measurements						
TG, mmol/L	0.71 [0.57, 0.90]	0.68 [0.56, 0.92]	0.71 [0.57, 0.92]	0.72 [0.57, 0.94]	0.70 [0.57, 0.92]	0.524
TC, mmol/L	4.64 (0.84)	4.69 (0.92)	4.64 (0.80)	4.69 (0.87)	4.67 (0.86)	0.767
LDL-C, mmol/L	2.80 (0.65)	2.83 (0.63)	2.80 (0.62)	2.83 (0.64)	2.82 (0.63)	0.847
HDL-C, mmol/L	1.57 (0.28)	1.59 (0.29)	1.57 (0.29)	1.60 (0.29)	1.59 (0.29)	0.372
FPG, mmol/L	4.96 (0.34)^a^	4.93 (0.38)^ab^	4.91 (0.34)^ab^	4.87 (0.38)^b^	4.91 (0.36)	**0.022**
FINs, pmol/L	47.9 [34.7, 68.5]	47.1 [33.0, 65.8]	47.0 [32.9, 64.2]	45.4 [32.8, 59.5]	46.7 [33.1, 63.8]	0.328
HOMA-IR	1.49 [1.08, 2.23]	1.46 [1.02, 2.11]	1.48 [1.02, 2.06]	1.41 [1.01, 1.89]	1.47 [1.02, 2.02]	0.174
CMR score	0.11 (2.96)	−0.05 (3.11)	0.07 (3.11)	−0.17 (2.86)	−0.02 (3.02)	0.642
20mSRT, laps	25.0 (14.4)^a^	26.6 (13.7)^ab^	27.5 (13.7)^b^	28.6 (14.1)^b^	27.1 (13.9)	**0.020**
Time of MVPA, n (%)						**<0.001**
More than 1 h/day	109 (49.8)^a^	178 (44.8)^a^	215 (50.6)^a^	211 (60.6)^b^	713 (51.3)	
Less than 1 h/day	110 (50.2)^a^	219 (55.2)^a^	210 (49.4)^a^	137 (39.4)^b^	676 (48.7)	

BFP, body fat percentage; BMI, body mass index; CMR: cardiometabolic risk; DBP, diastolic blood pressure; FINs, fasting insulin; FPG, fasting plasma glucose; GDR: global dietary recommendations; HDL-C, high-density lipoprotein cholesterol; HOMA-IR, homeostatic model assessment for insulin resistance; LDL-C, low-density lipoprotein cholesterol; MVPA: moderate-to-vigorous physical activity; SBP, systolic blood pressure; TC, total cholesterol; TG, triglycerides; WHR, waist-to-hip ratio; WHtR, waist-to-height ratio; 20mSRT: 20 - meter shuttle run test.

a, b: The values with different superscript letters in a row mean significantly different (*p* < 0.05) between quantiles. Bold values indicate statistical significance.

Dietary characteristics (Table 2) revealed marked variations in food consumption patterns. The consumption of healthy food items increased significantly with higher GDR scores. For example, the proportion of children consuming whole grains rose from 20.1% in Q1 to 64.9% in Q4, with significant pairwise differences across most quantiles (*p* < 0.05). Similar increasing trends were observed for pulses, vitamin A-rich vegetables, dark green leafy vegetables, other vegetables, citrus fruits, and other fruits (all *p* < 0.001). Conversely, the consumption of unhealthy food items declined markedly with higher GDR scores. For instance, the prevalence of soft drink intake decreased from 41.6% in Q1 to just 3.7% in Q4, and significant differences were also observed for baked sweets, processed meats, deep-fried foods, fast food, and packaged salty snacks (all *p* < 0.001). Overall, the GDR score itself increased stepwise across quantiles (7.1 ± 1.2 in Q1 vs. 13.9 ± 1.1 in Q4, *p* < 0.001).

**Table 2:** Dietary characteristics of participants across quantiles of GDR score.

Variables	Q1	Q2	Q3	Q4	Overall	*P*
*n*	219	397	425	348	1,389	
Healthy food items consumed, *n* (%)						
Whole grains	44 (20.1)^a^	110 (27.7)^ab^	147 (34.6)^b^	226 (64.9)^c^	527 (37.9)	**<0.001**
Pulses	55 (25.1)^a^	119 (30.0)^ab^	153 (36.0)^b^	185 (53.2)^c^	512 (36.9)	**<0.001**
Nuts and seeds	37 (16.9)^a^	71 (17.9)^a^	93 (21.9)^ab^	105 (30.2)^b^	306 (22.0)	**<0.001**
Vitamin A-rich orange vegetables	55 (25.1)^a^	146 (36.8)^b^	206 (48.5)^c^	265 (76.1)^d^	672 (48.4)	**<0.001**
Dark green leafy vegetables	91 (41.6)^a^	191 (48.1)^a^	264 (62.1)^b^	310 (89.1)^c^	856 (61.6)	**<0.001**
Other vegetables	98 (44.7)^a^	220 (55.4)^a^	298 (70.1)^b^	306 (87.9)^c^	922 (66.4)	**<0.001**
Vitamin A-rich fruits	59 (26.9)^a^	108 (27.2)^a^	134 (31.5)^a^	185 (53.2)^b^	486 (35.0)	**<0.001**
Citrus	72 (32.9)^a^	158 (39.8)^ab^	194 (45.6)^b^	232 (66.7)^c^	656 (47.2)	**<0.001**
Other fruits	123 (56.2)^a^	244 (61.5)^a^	331 (77.9)^b^	307 (88.2)^c^	1,005 (72.4)	**<0.001**
Unhealthy food items consumed, *n* (%)						
Soft drinks (sodas)	91 (41.6)^a^	92 (23.2)^b^	27 (6.4)^c^	13 (3.7)^c^	223 (16.1)	**<0.001**
Baked/grain-based sweets	115 (52.5)^a^	141 (35.5)^b^	130 (30.6)^b^	71 (20.4)^c^	457 (32.9)	**<0.001**
Other sweets	151 (68.9)^a^	164 (41.3)^b^	108 (25.4)^c^	60 (17.2)^d^	483 (34.8)	**<0.001**
Processed meat*	145 (66.2)^a^	145 (36.5)^b^	98 (23.1)^c^	42 (12.1)^d^	430 (31.0)	**<0.001**
Unprocessed red meat	118 (53.9)^a^	166 (41.8)^b^	153 (36.0)^bc^	110 (31.6)^c^	547 (39.4)	**<0.001**
Deep fried food	104 (47.5)^a^	120 (30.2)^b^	67 (15.8)^c^	23 (6.6)^d^	314 (22.6)	**<0.001**
Fast food & Instant noodles	99 (45.2)^a^	97 (24.4)^b^	58 (13.6)^c^	24 (6.9)^d^	278 (20.0)	**<0.001**
Packaged ultra-processed salty snacks	82 (37.4)^a^	96 (24.2)^b^	44 (10.4)^c^	16 (4.6)^d^	238 (17.1)	**<0.001**
GDR score, point	7.1 (1.2)^a^	9.5 (0.5)^b^	11.4 (0.5)^c^	13.9 (1.1)^d^	10.8 (2.4)	**<0.001**

* This item is scored 2 points.

GDR: global dietary recommendations.

a, b, c, d: The values with different superscript letters in a row mean significantly different (*p* < 0.05) between quantiles. Bold values indicate statistical significance.

### 3.2 Associations between GDR score and cardiometabolic indicators

The associations between GDR score and cardiometabolic indicators were assessed using two models (as presented in Table 3): an unadjusted model (Model 1) and a model adjusted for age, sex and MVPA (Model 2). In Model 1, higher GDR scores were inversely associated with BMI Z-score (*β* = −0.029, *p =* 0.044), WC (*β* = −0.161, *p =* 0.035), WHR (*β* = −0.002, *p =* 0.003), WHtR (*β* = −0.001, *p =* 0.024), FPG (*β* = −0.007, *p =* 0.047), FINS (*β* = −0.011, *p =* 0.048) and HOMA-IR (*β* = −0.013, *p =* 0.028), while positively linked to CRF (*β* = 0.405, *p =* 0.006). After adjustment for covariates in Model 2, GDR score remained significantly associated with FINS (*β* = −0.013, *p =* 0.023), HOMA-IR (*β* = −0.014, *p =* 0.019), and CRF (*β* = 0.441, *p =* 0.003). Additionally, GDR score was negatively associated with CMR score (*β* = −0.074, *p =* 0.030) in Model 2.

**Table 3:** Associations between GDR and cardiometabolic indicators.

Variables	Model 1		Model 2	
	β (95% CI)	*p*	β (95% CI)	*p*
BMI	−0.056 (−0.112, 0.001)	0.055	−0.042 (−0.098, 0.015)	0.150
BMI Z score	−0.029 (−0.056, −0.001)	**0.044**	−0.020 (−0.048, 0.007)	0.150
BFP	−0.093 (−0.264, 0.080)	0.287	−0.111 (−0.284, 0.065)	0.213
Waist circumference	−0.161 (−0.310, −0.011)	**0.035**	−0.108 (−0.255, 0.040)	0.153
WHR	−0.002 (−0.003, −0.001)	**0.003**	−0.001 (−0.002, 0.00001)	0.052
WHtR	−0.001 (−0.002, −0.0001)	**0.024**	−0.001 (−0.002, 0.0003)	0.154
SBP	−0.165 (−0.372, 0.043)	0.118	−0.131 (−0.340, 0.080)	0.223
DBP	0.043 (−0.119, 0.203)	0.604	0.050 (−0.114, 0.213)	0.550
FPG	−0.007 (−0.014, −0.0001)	**0.047**	−0.003 (−0.01, 0.004)	0.377
FINS	−0.011 (−0.022, −0.0001)	**0.048**	−0.013 (−0.024, −0.002)	**0.023**
HOMA-IR	−0.013 (−0.025, −0.002)	**0.028**	−0.014 (−0.026, −0.002)	**0.019**
TG	0.003 (−0.004, 0.011)	0.398	0.001 (−0.007, 0.009)	0.762
TC	−0.002 (−0.020, 0.016)	0.860	−0.004 (−0.022, 0.015)	0.678
HDL-C	0.004 (−0.002, 0.010)	0.199	0.005 (−0.001, 0.011)	0.098
LDL-C	−0.001 (−0.014, 0.013)	0.920	−0.003 (−0.016, 0.011)	0.677
CMR score	−0.064 (−0.129, 0.002)	0.054	−0.074 (−0.139, −0.006)	**0.030**
20mSRT	0.405 (0.115, 0.698)	**0.006**	0.441 (0.152, 0.732)	**0.003**

Model 1: unadjusted model.

Model 2: additionally adjusted for age, sex and MVPA on the basis of model 1.

BFP, body fat percentage; BMI, body mass index; CMR: cardiometabolic risk; DBP, diastolic blood pressure; FINs, fasting insulin; FPG, fasting plasma glucose; GDR: global dietary recommendations; HDL-C, high-density lipoprotein cholesterol; HOMA-IR, homeostatic model assessment for insulin resistance; LDL-C, low-density lipoprotein cholesterol; SBP, systolic blood pressure; TC, total cholesterol; TG, triglycerides; WHR, waist-to-hip ratio; WHtR, waist-to-height ratio; 20mSRT: 20 - meter shuttle run test. Bold values indicate statistical significance.

### 3.3 Stratified analysis by sex

The associations between GDR score and cardiometabolic indicators were further stratified by sex (Table 4). The interaction terms between sex and GDR score were significant for BMI (*p =* 0.020), BMI Z-score (*p =* 0.037), and BFP (*p =* 0.025), indicating that the associations between GDR score and these indicators differed by sex. Among boys, GDR score was not significantly associated with most cardiometabolic indicators, except for a positive association with 20mSRT (*β* = 0.451, *p =* 0.042). In contrast, among girls, GDR score was significantly associated with BMI (*β* = −0.108, *p =* 0.005), BMI Z-score (*β* = −0.049, *p =* 0.008), BFP (*β* = −0.322, *p =* 0.008), WC (*β* = −0.225, *p =* 0.023), WHtR (*β* = −0.002, *p =* 0.014), FINS (*β* = −0.021, *p =* 0.012), HOMA-IR (*β* = −0.022, *p =* 0.010), and CMR score (*β* = −0.126, *p =* 0.014).

**Table 4:** Associations between GDR and cardiometabolic indicators stratified by sex.

Variables	Boys		Girls		*P* for interaction
	β (95% CI)	*p*	β (95% CI)	*p*	
BMI	0.016 (−0.068, 0.099)	0.716	−0.108 (−0.183, −0.033)	**0.005**	**0.020**
BMI *Z* score	0.004 (−0.037, 0.046)	0.839	−0.049 (−0.085, −0.013)	**0.008**	**0.037**
BFP	0.082 (−0.167, 0.340)	0.524	−0.322 (−0.557, −0.082)	**0.008**	**0.025**
Waist circumference	−0.001 (−0.218, 0.222)	0.997	−0.225 (−0.419, −0.032)	**0.023**	0.094
WHR	−0.001 (−0.002, 0.001)	0.324	−0.001 (−0.003, 0.0001)	0.073	0.515
WHtR	0.0001 (−0.001, 0.002)	0.876	−0.002 (−0.003, −0.0003)	**0.014**	0.073
SBP	−0.084 (−0.364, 0.198)	0.558	−0.188 (−0.504, 0.131)	0.246	0.672
DBP	0.108 (−0.117, 0.331)	0.347	−0.023 (−0.267, 0.215)	0.852	0.467
FPG	−0.001 (−0.011, 0.009)	0.796	−0.006 (−0.016, 0.003)	0.208	0.526
FINS	−0.007 (−0.022, 0.009)	0.413	−0.021 (−0.037, −0.005)	**0.012**	0.190
HOMA IR	−0.008 (−0.025, 0.008)	0.349	−0.022 (−0.039, −0.006)	**0.010**	0.183
TG	0.004 (−0.007, 0.016)	0.456	−0.002 (−0.013, 0.009)	0.687	0.458
TC	0.004 (−0.02, 0.027)	0.758	−0.014 (−0.043, 0.015)	0.330	0.482
HDL-C	0.004 (−0.004, 0.012)	0.357	0.007 (−0.002, 0.016)	0.153	0.632
LDL-C	0.003 (−0.015, 0.021)	0.730	−0.011 (−0.031, 0.009)	0.293	0.380
CMR score	−0.024 (−0.112, 0.067)	0.599	−0.126 (−0.224, −0.024)	**0.014**	0.130
20mSRT	0.451 (0.018, 0.886)	**0.042**	0.488 (0.120, 0.862)	**0.010**	0.802

Model adjusted for age, sex and MVPA.

BFP, body fat percentage; BMI, body mass index; CMR: cardiometabolic risk; DBP, diastolic blood pressure; FINs, fasting insulin; FPG, fasting plasma glucose; GDR: global dietary recommendations; HDL-C, high-density lipoprotein cholesterol; HOMA-IR, homeostatic model assessment for insulin resistance; LDL-C, low-density lipoprotein cholesterol; SBP, systolic blood pressure; TC, total cholesterol; TG, triglycerides; WHR, waist-to-hip ratio; WHtR, waist-to-height ratio; 20mSRT: 20 - meter shuttle run test. Bold values indicate statistical significance.

### 3.4 Mediating effect of CRF

**Figure 2:**
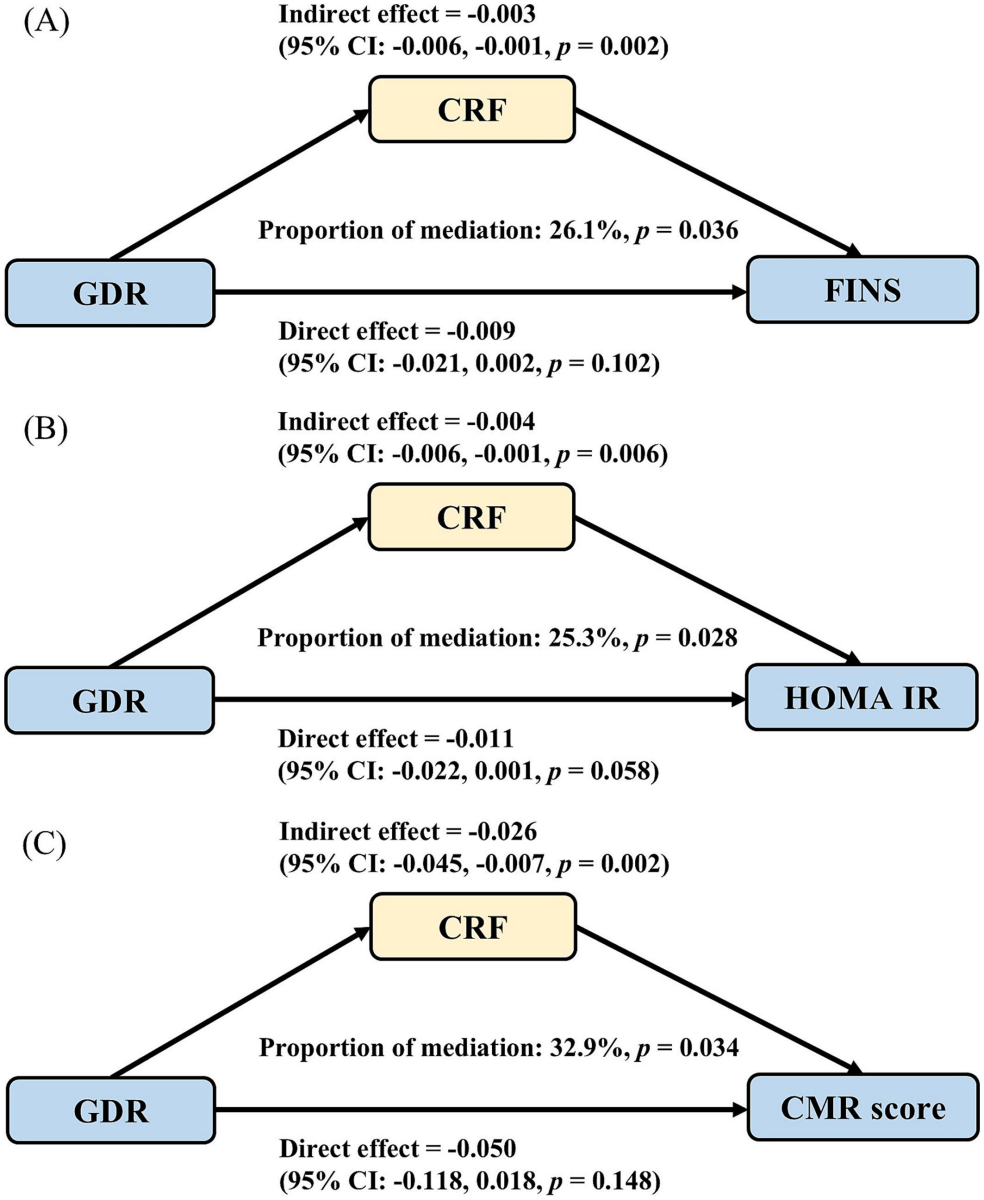
The mediating role of cardiorespiratory fitness in associations between GDR and cardiometabolic risk factors. **(A)** The mediation between GDR score and FINS; **(B)** The mediation between GDR score and HOMA IR; **(C)** The mediation between GDR score and CMR-score. CMR: cardiometabolic risk; CRF: cardiorespiratory fitness; FINS, fasting insulin; GDR: global dietary recommendations; HOMA-IR, homeostatic model assessment for insulin resistance.

We explored the mediating effect of CRF between GDR score and cardiometabolic indictors (Figure 2). CRF mediated 26.1% (*p =* 0.036, Figure 2A) and 25.3% (*p =* 0.028, Figure 2B), of the association between GDR score and FINS and HOMA-IR, and we found the largest mediating proportion of the association between GDR score and CMR score (mediation effect: 32.9%, *p =* 0.034, Figure 2C).

## 4 Discussion

In this study, we found that higher diet quality, characterized by greater adherence to global dietary recommendations, was associated with more favorable cardiometabolic profiles, including insulin resistance (HOMA-IR), FINS, and better overall metabolic health, particularly among girls. Furthermore, CRF was found to mediate the relationship between diet quality and CMR indicators, suggesting that improving physical fitness might enhance the benefits of a healthy diet on childhood cardiometabolic health.

The GDR score, which captures adherence to global dietary recommendations, provides a valuable tool for quantifying diet quality and its impact on health outcomes in diverse populations (19, 39–41). Unlike other dietary assessment methods that may be burdensome or complex (17), the DQQ used in our study offers a low-burden approach to evaluating dietary adherence. Our findings align with existing literature suggesting that higher diet quality is associated with better cardiometabolic health outcomes (11, 14). Poor diet quality, characterized by excessive intake of sugar, processed foods, and saturated fats, has been strongly linked to obesity, type 2 diabetes, and cardiovascular disease (42). Our study reinforces these findings by demonstrating that children with higher GDR scores exhibited lower BMI Z-score, waist circumference, FINS, HOMA-IR and other metabolic markers. These results highlight the importance of promoting high-quality dietary patterns in childhood to improve future cardiometabolic risks. The validation of the DQQ in Chinese children further strengthens its utility as a reliable instrument for monitoring diet quality in this demographic.

Interestingly, our stratified analysis by sex revealed that the associations between diet quality and cardiometabolic indicators were stronger among girls than boys. Siddiqui et al. noted more pronounced diet-blood pressure associations in boys within the Generation R Study, a prospective population-based cohort in Rotterdam (10), while our data revealed girls exhibited greater reductions in adiposity and insulin resistance with higher GDR scores. This discrepancy may reflect cultural or behavioral factors, such as girls’ greater adherence to dietary guidelines or earlier maturation influencing metabolic responses. One possible explanation for this sex difference is physiological variations in insulin sensitivity and fat distribution before puberty (43). Studies suggest that girls experience a greater decline in insulin sensitivity during early adolescence, making them more susceptible to dietary influences (44). Future studies should explore these sex-specific pathways in greater detail to tailor dietary and physical activity interventions accordingly.

CRF has been widely recognized as a key predictor of cardiometabolic health in children (45). Previous research has shown that higher CRF is associated with lower metabolic risk, including reduced HOMA-IR, TG, and higher HDL-C (46). This study further confirmed that CRF mediated the associations between GDR score and CMR factors, particularly for FINS, HOMA-IR, and the composite CMR score. Our findings are consistent with intervention studies showing that physical exercise interventions reduced HOMA-IR and FINS in children, although fasting glucose often remains unchanged (47).

Physiologically, higher CRF is associated with improved insulin sensitivity (48), reduced inflammation (49), and enhanced lipid metabolism (50, 51). However, the observed mediation was partial, with CRF explaining approximately 25.3 to 32.9% of the association between diet quality and cardiometabolic outcomes. The partial mediation suggests additional pathways are likely responsible for the remaining 67 to 75% of the relationship, such as gut microbiota modulation or epigenetic regulation, for instance, fibre-rich diets increase short-chain fatty acid production, which enhances insulin signaling (52). Likewise, certain nutrients can modify DNA methylation and histone acetylation patterns, influencing gene expression involved in glucose metabolism and lipid regulation (53–55). Given that these pathways respond directly to dietary components and are independent of physical fitness, it is plausible that their contribution to cardiometabolic regulation may exceed that of CRF alone. Therefore, future studies should not only include CRF as a mediator but also integrate measures of gut microbiota composition and epigenetic markers to clarify their respective roles.

Despite its strengths, including the large sample size and the use of validated tools for diet and comprehensive cardiometabolic measurements, this study has several limitations. First, the cross-sectional design limits our ability to infer causality between diet quality, CRF, and cardiometabolic outcomes. Although mediation analysis suggested a potential pathway linking diet quality, CRF, and cardiometabolic markers, longitudinal or experimental studies are needed to confirm temporal precedence and causality. Second, dietary data were self-reported, potentially introducing recall bias. Thirdly, a potential issue is whether the CRF assessed through the 20mSRT is influenced by body size and composition. However, the 20mSRT remains a suitable, practical, and widely recommended method for large-scale assessments in the pediatric population (56).

In conclusion, this study provides evidence that higher diet quality is associated with better cardiometabolic health in Chinese children, with CRF playing a potential mediating role. These findings support the need for comprehensive health promotion strategies that integrate both healthy eating and physical activity to improve childhood obesity and other CMR factors. Further research is needed to better understand the mechanisms underlying these relationships and to explore their long-term implications for adult health.

## Data Availability

The raw data supporting the conclusions of this article will be made available by the authors, without undue reservation.

## Glossary

ANOVA: Analysis of Variance
BFP: Body Fat Percentage
BMI: Body Mass Index
CMR: Cardiometabolic Risk
CRF: Cardiorespiratory Fitness
DBP: Diastolic Blood Pressure
DQQ: Diet Quality Questionnaire
FINS: Fasting Insulin
FPG: Fasting Plasma Glucose
GDR: Global Dietary Recommendations
HDL-C: High-Density Lipoprotein Cholesterol
HOMA-IR: Homeostasis Model Assessment for Insulin Resistance
IQR: Interquartile Range
LDL-C: Low-Density Lipoprotein Cholesterol
MVPA: Moderate-to-Vigorous Physical Activity
SBP: Systolic Blood Pressure
TC: Total Cholesterol
TG: Triglycerides
WHR: Waist-to-Hip Ratio
WHtR: Waist-to-Height Ratio
20mSRT: 20-Meter Shuttle Run Test
