# Supplementary material for: The associations of diet quality and cardiometabolic indicators in children and the mediation role of cardiorespiratory fitness
Source: Front Nutr. 2025 Oct 23;12:1632493. doi: 10.3389/fnut.2025.1632493 (PMC12588837; doi:10.3389/fnut.2025.1632493)
Supplement: Supplementary file 1 [file Table_1.docx]

**Supplementary materials**

**Table S1. Comparation of the baseline characteristic between children included (n=1389) and excluded (n=251) in analyses.**

|  | **Included in analyses** | **Excluded in analyses** | ***P*** |
| --- | --- | --- | --- |
| n | 1389 | 251 |  |
| Sex, n (%) |  |  | 0.550 |
| Boys | 732 (52.7) | 138 (55.0) |  |
| Girls | 657 (47.3) | 113 (45.0) |  |
| Mean age (SD), year | 8.48 (0.29) | 8.48 (0.31) | 0.932 |
| Mean height (SD), cm | 132.4 (5.7) | 132.3 (6.1) | 0.918 |
| Mean weight (SD), kg | 29.3 (6.2) | 28.1 (6.6) | 0.195 |
| Mean waist circumference (SD), cm | 57.6 (7.0) | 56.6 (7.3) | 0.067 |
| Mean hip circumference (SD), cm | 70.5 (6.6) | 69.6 (6.9) | 0.064 |
| Mean WHR (SD) | 0.82 (0.05) | 0.82 (0.05) | 0.644 |
| Mean WHtR (SD) | 0.43 (0.05) | 0.43 (0.05) | 0.123 |
| Mean BFP (SD), % | 20.0 (7.9) | 19.2 (8.0) | 0.327 |
| Mean BMI Z score (SD) | 0.19 (1.28) | 0.10 (1.29) | 0.185 |
| Mean BMI (SD), kg/m^2^ | 16.6 (2.6) | 16.1 (2.7) | 0.132 |
| Mean SBP (SD), (mmHg) | 101.6 (10.4) | 101.3 (11.4) | 0.562 |
| Mean DBP (SD), (mmHg) | 63.3 (7.6) | 62.7 (7.8) | 0.233 |
| Mean TG (SD), (mmol/L) | 0.79 (0.34) | 0.78 (0.34) | 0.748 |
| Mean TC (SD), (mmol/L) | 4.67 (0.86) | 4.74 (1.01) | 0.171 |
| Mean LDL-C (SD), (mmol/L) | 2.82 (0.63) | 2.91 (0.76) | 0.134 |
| Mean HDL-C (SD), (mmol/L) | 1.59 (0.29) | 1.55 (0.32) | 0.536 |
| Mean FPG (SD), (mmol/L) | 4.91 (0.36) | 4.89 (0.39) | 0.302 |
| Mean FINs (SD), (pmol/L) | 52.7 (29.7) | 62.9 (93.1) | 0.099 |
| Mean 20mSRT (SD), laps | 27.1 (13.9) | 27.2 (13.2) | 0.913 |

*Abbreviations*: BFP, body fat percentage; BMI, body mass index; DBP, diastolic blood pressure; FINs, fasting insulin; FPG, fasting plasma glucose; HDL-C, high-density lipoprotein cholesterol; HOMA-IR, homeostatic model assessment for insulin resistance; LDL-C, low-density lipoprotein cholesterol; SBP, systolic blood pressure; TC, total cholesterol; TG, triglycerides; WHR, waist-to-hip ratio; WHtR, waist-to-height ratio; 20mSRT: 20 - meter shuttle run test.

**
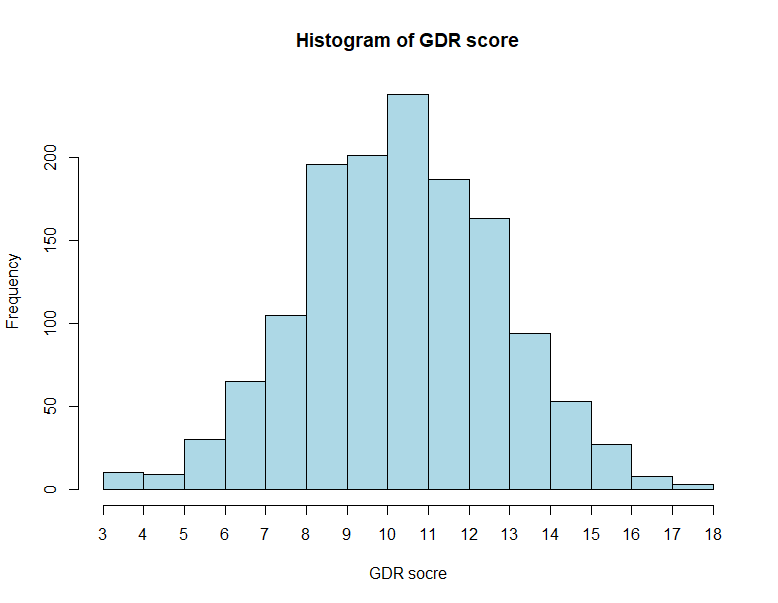
**

**Figure S1. Histogram of GDR score.**
